# The Key Role of Warm and Cold Ischemia in Uterus Transplantation: A Review

**DOI:** 10.3390/jcm8060760

**Published:** 2019-05-29

**Authors:** Antoine Tardieu, Ludivine Dion, Vincent Lavoué, Pauline Chazelas, Pierre Marquet, Pascal Piver, Camille Sallée, Yves Aubard, Chantal Barin-Le Guellec, Frederic Favreau, Tristan Gauthier

**Affiliations:** 1 INSERM, INSERM Unit U1248, University of Medicine, 87000 Limoges, France; antoine.tardieu@hotmail.fr (A.T.); pierre.marquet@unilim.fr (P.M.); pascal.piver@unilim.fr (P.P.); yves.aubard@unilim.fr (Y.A.); chantal.barin-leguellec@univ-tours.fr (C.B.-L.G.); 2 Department of Obstetrics and Gynecology, Limoges University Hospital Center, 87000 Limoges, France; camille.sallee@gmail.com; 3 Department of Obstetrics and Gynecology, Rennes University Hospital Center, 35000 Rennes, France; dion.ludivine@orange.fr (L.D.); vincent.lavoue@gmail.com (V.L.); 4 INSERM, INSERM Unit 1242, University of Medicine, 35000 Rennes, France; 5 University of Limoges, Faculty of Medicine, EA 6309, Maintenance myélinique et neuropathies périphériques, 87000 Limoges, France; pauline.chazelas@gmail.com (P.C.); frederic.favreau@chu-limoges.fr (F.F.); 6 Limoges University Hospital Center, Laboratory of Biochemistry and Molecular Genetics, 87000 Limoges, France; 7 University of Tours, Faculty of Medicine, 37000 Tours, France

**Keywords:** ischemia, uterus, transplantation, deceased donor

## Abstract

*Introduction*: Uterus transplantation (UTx) is a promising treatment for uterine infertility that has resulted in several births since 2014. Ischemia is a key step in organ transplantation because it may lead to changes jeopardizing graft viability. *Method*: We performed a systematic review of animal and human studies relating to uterine ischemia. *Results*: We retained 64 studies published since 2000. There were 35 studies in animals, 24 in humans, and five literature reviews. Modest preliminary results in large animals and humans are limited but encouraging. In small animals, pregnancies have been reported to occur after 24 h of cold ischemia (CI). In ewes, uterine contractions have been detected after 24 h of CI. Furthermore, it has been shown in animals that uterine tolerance to CI and to warm ischemia (WI) can be increased by pharmacological products. In women, mean CI time in studies of births from uteri obtained from live donors was between 2 h 47 min and 6 h 20 min from a deceased donor; with only one birth in this case. Muscle contractions have also been demonstrated in myometrial samples from women, after six or more hours of CI. *Conclusion*: The uterus seems to be able to tolerate a prolonged period of CI, of at least six hours. Studies of the ischemia tolerance of the uterus and ways to improve it are essential for the development of UTx, particularly for procedures using grafts from deceased donors.

## 1. Introduction

Uterus transplantation (UTx) is an interesting alternative to surrogacy and adoption in the case of uterine factor infertility and motherhood wishing. It has the advantage over these other approaches of allowing the woman to be both the genetic mother and the legal bearer of the child. There is a tremendous demand for treatments for uterine infertility, and UTx is a source of great hope for many of these women [1].

The first UTx was performed in 2000. The uterus had to be removed after only three months, due to graft necrosis [2]. An increasing number of studies in animals and humans are directly or indirectly focusing on UTx, with the aim of increasing success rates for this procedure. The results of these studies led to the first human birth after UTx in 2014; reported by the team of Brännström in Sweden [3,4]. According to the first state-of-the-art meeting on UTx organized by the International Society of Uterus Transplantation (ISTUx) in October 2018, 52 UTx procedures have been carried out around the world [5].

During organ transplantation, ischemia at body temperature (“warm ischemia”) or during the ex vivo storage of grafts in hypothermic conditions (“cold ischemia”), may lead to metabolic and histologic lesions that are amplified during reperfusion of the organ, potentially resulting in an acute or chronic loss of function [6]. Cold ischemia (CI) is defined as the period of graft storage ex vivo in hypothermic conditions, beginning with the perfusion of the organ with hypothermic flushing solution and extending to vascular anastomosis in the recipient. Warm ischemic occurs when vascular flow in the donor is interrupted (by arterial clamping) before the perfusion of the graft with graft storage solution (first phase), but also during vascular anastomosis (second phase). This second phase of warm ischemia (WI) is followed by reperfusion. With living donors, the CI stage is reduced to a few hours at most. The capacity of organs to tolerate CI and to remain functional depends on the type of organ: generally, no more than 4 h for the heart, but up to 24 h for kidneys. This tolerance is known for the three most commonly transplanted organs (kidney, heart and liver), but the maximum time for which uterine grafts can be stored in cold conditions remains undetermined, and the optimal composition of graft storage medium also remains unknown for this organ. Studies of CI tolerance and of adaptation of the storage medium are therefore of the utmost importance, to optimize UTx from brain-dead donors [7].

The principal objective of this study was to review the date about tolerance of ischemia of uterus in the field of UTx.

## 2. Materials and Methods

We included studies relating to uterine ischemia, with or without reperfusion, in the context of UTx. Studies on humans, non-human primates and non-primates were included. We excluded studies dealing with uterine ischemia during pregnancy.

Original research studies, case reports, meta-analyses, and reviews published in English or French were eligible for inclusion.

We searched the PubMed bibliographic database for publications over a period of 19 years, extending from January 2000 to May 2019.

We used diverse keywords, together or in isolation, to obtain the maximum number of relevant results during the literature review. We used the following keywords: “uterine transplantation”, “ischaemic”, “ischemia”, “tolerance”, “warm ischemia”, “cold ischemia”, “ischaemic preservation”, “allogeneic transplantation”, “uterine autotransplantation”, “ischemia reperfusion”, “pig”, “sheep”, “ewe”, “rabbit”, “mouse”, “rat”, “non-human primate”, and “human”. We minimized the possibility of duplication by downloading all the key fields for each study, including the unique identification codes (e.g., PMID), the digital object identification number (DOI), the number of the clinical trial (www.clinicaltrials.gov), the abstract, and the keywords. The abstracts of the selected studies were then examined to determine whether they met the predefined inclusion and exclusion criteria, and all non-pertinent studies were eliminated. Studies meeting all the criteria for inclusion and none of those for exclusion were selected for an examination of the full text and data extraction. We also searched for unpublished journal articles and for conference proceedings. Clinical trial registers were searched for unpublished trials and trials underway. The references cited in the selected publications were also examined, to identify additional relevant studies.

## 3. Results

A literature search identified 288 articles relating to uterus transplantation: 61 of these articles dealt with uterine ischemia: 24 focused on uterine ischemia in humans, seven in non-human primates and 28 were performed in non-primates. The five remaining studies were literature reviews.

## 4. Experimental and Clinical Studies

Most of these studies evaluating uterine ischemia tolerance were performed in a context of UTx or simple arterial clamping. The organs were thus subject to CI and WI, respectively, followed by reperfusion. The studies of uterine ischemia identified included a few studies on humans, but a large number of studies in many species of animals. Multiple outcome measures can be used to assess uterine ischemia tolerance. Histological, biochemical (e.g., pH, PO_2_, PCO_2_), and macroscopic (uterine coloration, spontaneous or induced uterine contractions, menstruation after transplantation) criteria can be used, but the best criterion for uterine ischemia tolerance is the occurrence of a pregnancy leading to a birth. Autologous grafts and syngeneic grafts can be used to overcome the problem of immune reactions to the graft; these approaches are currently the best study models for ischemia-reperfusion. Allogeneic grafts can be used to take the immune alloreaction into account and to study immunosuppression.

### 4.1. Animal Studies

#### 4.1.1. Impact of CI Time in the Context of UTx

In 2003, El-Akouri et al. performed syngeneic heterotopic transplantations in mice with various CI times (24 or 48 h) [8]. The CI time was 24 h in the first group and 48 h in the second group. The uteri were stored in a preservation fluid of the University of Wisconsin solution (UW) type or in 0.9% NaCl. No significant differences in morphological or microscopic appearance were observed between the two storage fluids after 24 h of CI. By contrast, after 48 h of CI in UW, signs of degeneration or necrosis (changes in color, with a decrease in arterial pulsation) were observed in the graft two weeks after transplantation. However, spontaneous contractions and contractions induced by prostaglandin F_2α_ (PGF_2α_) were conserved after 24 or 48 h of ischemia, regardless of the conservation fluid used, but these contractions were weaker after storage in 0.9% NaCl than after storage in UW. Embryos were transplanted into six mice implanted with uteri that had been subjected to 24 h of CI in the UW uterine storage solution. Pregnancies were established in five of these mice; 25 births, with no abnormalities, were recorded. No embryos were transplanted into the group of mice receiving uteri subjected to 48 h of ischemia. In the same year, El-Akouri et al. obtained pregnancies and births after syngeneic heterotopic transplantation, but after only short periods of CI (about 20 min) [9]. In a study performed in rats in 2011, Wranning et al. [10] obtained multiple pregnancies (*n* = 11) and births (*n* = 6) after syngeneic transplantation with a CI time of 120 min.

The choice of animal model is crucial for rapid clinical development. Pigs and ewes have yielded pertinent results, given their close anatomical similarities to humans. In 2006, Wranning et al. [11] performed 19 autologous transplantations in a pig model of uterus transplantation. Only seven of these transplantations were considered successful. The mean (±SD) CI time was 90 min (±28). The graft was perfused with Ringer’s lactate solution. The authors found no signs of severe biochemical ischemic lesions such as acidosis (pH, lactate), chronic hypoxia (lactate, pCO_2_/pO_2_), oxidative stress (substances reacting with thiobarbituric acid) on venous samples (jugular and uterine veins) collected 20, 60 or 100 min after reperfusion, or of severe histological lesions on uterine biopsy specimens collected after 60 min of reperfusion, and just before the end of the intervention. In 2008, Dahm-Kahler et al. and Wranning et al. [12,13] performed autologous transplantation in ewes with CI and WI times of about 60 min each (seven procedures in the study by Dahm-Kahler et al. and 10 in that by Wranning et al.). The authors observed no macroscopic, biochemical or histological signs of severe ischemia lesions. In addition, Wranning et al. compared Perfadex^®^ with Ringer’s lactate solution as the storage fluid. They observed fewer secondary ischemia lesions following storage in Perfadex^®^ [13]. In 2010, Wranning et al. [14] obtained spontaneous pregnancies and births (*n* = 2) following 14 autologous transplantations in ewes, with the preservation of the ovarian vascular system, after a CI time of one hour. In 2013, Wei et al. [15] performed 10 autologous transplantations in ewes with a median (range) CI time of 179 min (130–270) and a WI time of 10 min (8–15). They observed only subtle signs of inflammation in their histological analysis of a single uterus, three months after transplantation. They noted an increase in circulating lactate concentration and pCO_2_/pO_2_ ratio (a sign of hypoxia) at the end of the period of WI, during the period of CI and during reperfusion, followed by a gradual return to normality. Transrectal ultrasound monitoring of the uterus showed the ovaries, a myometrium of normal appearance, and an absence of necrosis or edema. Vascular flow also appeared normal on a Doppler scan [15].

In all these studies, the total duration of ischemia may be considered short. Our team evaluated the tolerance of the ovine uterus to longer ischemia times in the context of autologous transplantation [16]. We performed 14 autologous transplantations in ewes, with CI times of 3 h or of 24 h with storage in Celsior^®^ at 4 °C. In the first group, the median (range) cold and warm ischemia times were 190 (180–205) and 70 (59–105) min, respectively. In the second group, these times were 1360 (1360–1456) and 56 min (52–71) min, respectively. For the 12 transplantations performed, we found no significant difference between the two groups in terms of apoptosis. Sixty seven percent of the uteri were contractile after 3 h of CI and 75% were contractile after 24 h of CI.

Recently, the Swedish team of Pr Brännström evaluated the impact of prolonged CI in a sheep model with reperfusion similarity. Thirteen sheep uteri were perfused during storage for 4 h (*n* = 6) or 48 h (*n* = 7) by a solution of IGL-1 followed by reperfusion for 48 h under normothermic conditions with an oxygenated recirculating perfusate containing growth factors and synthetic oxygen carriers. Biopsies performed in the 4 h group of CI indicated no significant edema in the myometrium or endometrium. Only the outer serous layer and luminal epithelial cells of the inner column were affected by reperfusion. However, a much faster and more severe reperfusion injury of all uterine layers was evident during the reperfusion experiment after 48 h of cold ischemia. Significant accumulation of lactate was measured in the perfusate with a subsequent decrease in pH [17].

Primates are the model of choice for animal experimentation of UTx, due to their close anatomical and physiological resemblance to humans. The first study of autologous UTx in baboons was performed in 2010 [18]. Ten autologous transplantations were performed, with a mean total ischemia time (WI + CI) of 2 h 51 min (±21 min), including one hour of WI. Nine of these transplantations were considered successful. The re-establishment of a biological ovarian cycle was observed in five females, the recommencement of menstruation was observed in only two of them and no spontaneous pregnancies had occurred after 10 months of follow-up. In the females in which the ovarian cycle was not re-established or was re-established late, a complete involution of the uterus was observed, with numerous adhesions. Johannesson et al. [19] also performed autologous transplantation in 16 female baboons. They used different anastomosis techniques. In the face of a high failure rate for the first interventions (group 1), a vascular surgeon was called in to modify the anastomosis technique (group 2). The median CI time was about 120 min in both groups, with about 53 min of WI. The biological ovarian cycle re-established itself in both groups, but menstruation recommenced only in group 2, with no pregnancies. Second-look laparotomy in these groups revealed the presence of numerous periovarian adhesions and a lack of tubular permeability, potentially accounting for the lack of pregnancies despite the return of menstruation. These studies demonstrate the importance of learning the surgical technique and its effects on the re-establishment of organ function.

In summary, pregnancies have been obtained in rodents after up to 24 h of CI. Few data are available concerning prolonged periods of CI in large animals. However, uterine contractions have been observed after 24 h of CI in ewes.

#### 4.1.2. Impact of Prolonged WI of the Uterus

Several studies have investigated the effect of prolonged WI on uterus viability.

Diaz-Garcia et al. [20] evaluated the tolerance of the rat uterus to ischemia, by performing syngeneic transplantations and varying the WI time. The CI time was identical in the two groups (about 120 min). By contrast, WI time ranged from 73 min (53–114) in one group (*n* = 10) to 314 min (298–359) in the other group. Uterine biopsies were performed on days three and six after transplantation. Clear macroscopic signs of necrosis (black coloration, firm consistency, and edema) were observed in half the individuals of the group with a longer WI time, versus only one in the first group. The histological results of the biopsies confirmed these results and highlighted the deleterious effects of prolonged WI on the uterus.

In primates, the first pregnancy after autologous transplantation dates back to 2012 [21]. After two autologous transplantations in cynomolgus macaques, a re-establishment of the hormone cycle was observed in the two females, after 5 h and 25 min of total ischemia in one female and 7 h 7 min of total ischemia in the other. However, only the second female displayed a return of menstruation and a physiological pregnancy followed by a birth. During the study, the WI times were long, due to the complexity of the vascular anastomoses, as in the previous study by these authors on the same model [22]. Adachi et al. in 2016 [23] and Kisu et al. in 2017 [24] focused on tolerance to WI induced in the uterus by simple arterial clamping. Using the macaque model (*n* = 6 in the study by Adachi et al. and *n* = 18 in the study by Kisu et al.), they found no evidence of a significant histological or biochemical (biochemical parameters of oxidative stress) difference after 4 or 8 h of WI followed by reperfusion. By contrast, they noted an absence of subsequent menstruation after 8 h of WI, but not after 4 h.

Overall, prolonged WI of the uterus is deleterious both histologically and biochemically. However, it is not possible to define a threshold for the moment, because the data obtained in animal models are heterogeneous.

#### 4.1.3. Uterine Ischemia and Allogeneic Transplantation

With a view to achieving conditions as close as possible to UTx in women, many allogeneic transplantations have been performed in animals, to integrate immunosuppression as a parameter. Diaz-Garcia et al. obtained pregnancies and births, with normal fetal development, in rats treated with tacrolimus after allogeneic transplantation (*n* = 10) [25]. In a study of allogeneic transplantation with tacrolimus treatment for immunosuppression, Saso et al. reported, in 2014 [26,27], that two of the five female rabbits undergoing transplantation were still alive one month after surgery. Despite repeated mating, no pregnancies occurred. In this study, the CI time was 116 min, the WI time was 155 min and the total ischemia time was 271 min [26]. Also in 2014, the same author obtained a pregnancy after the transfer of an embryo into the uterus of a female rabbit after autologous transplantation. However, this pregnancy ended in an early spontaneous abortion on day 18 [27]. In 2010, Ramirez et al. [28] obtained three pregnancies in ewes after the transfer of frozen blastocysts or fresh embryos, one of which ended in a birth. The immunosuppressant used was cyclosporine. One of the pregnancies was extrauterine and another ended in a spontaneous abortion. In this study, the mean CI and WI times were 60 and 40 min, respectively. Five allo-transplantations were performed with miniature swine in 2018 [29]. The success rate of transplantation was 100% and the 3-month survival was 80%. Mean CI duration was 54 min (±5.47) and 144 min (±15.1) for WI. Evaluation of transplant viability at 1 and 4 weeks after UTx by doppler ultrasound of uterine vessels was normal in 4 of 5 swine. An exploratory laparotomy was performed in 2 swine at 3 months and found a macroscopically viable uterus. On the other hand no pregnancy took place despite embryo transfer from UTx. In 2014, Kisu et al. [30] performed two allogeneic UTx between macaques. The total ischemia times were 4 h 16 min and 3 h 15 min, of which 1 h 13 min and 45 min, respectively, corresponded to CI. In the first case, menstruation recommenced after three months and then spontaneously stopped. Echographic evaluation showed the uterus to be unchanged in size but indicated that vascular flow in the left uterine artery had ceased. A histological analysis of the uterus found an absence of endometrial tissue in the intrauterine cavity and of interstitium in almost all the layers of the uterus wall. This wall displayed hyaline degeneration of no identifiable histological, macroscopic or echographic cause. By contrast, no sign of rejection was observed. In the second case, post-UTx amenorrhea was observed, with uterine atrophy on an ultrasound scan three months after transplantation. Histological analyses of the uterus revealed uterine atrophy, an absence of endometrial endothelium, and fibrosis with hemosiderosis and calcification. Immunohistochemistry revealed a nonspecific inflammatory response, with a mild infiltration of CD8-positive and CD20-positive lymphocytes into the interstitium, and no signs of rejection. There were, however, signs of bacterial infection.

All the animal studies resulting in live births, regardless of the type of UTx, are summarized in Table 1.

Overall, very few pregnancies have been obtained after allogeneic UTx in animals. Few data are available for prolonged ischemia.

#### 4.1.4. Pathophysiological Understanding of the Phenomenon of Ischemia

Several studies have focused on elucidating the pathophysiological mechanisms induced by uterine ischemia and its impact on uterine graft fate in animals. The objectives of these studies were to identify lesion markers for the evaluation of ischemia tolerance in the uterus and to limit these lesions by targeted pharmacological modulations or to make it possible to prolong CI, an essential step in the transplantation process.

The oxidative stress generated during ischemia is greatly amplified during reperfusion, corresponding to the reintroduction of oxygen. Reperfusion leads to the production of reactive oxygen species (ROS), which, as their name indicates, are highly reactive with all the major classes of biological molecules, with deleterious effects. They also stimulate inflammation by activating endothelial cells and through effects on myocytes, involving membrane and mitochondrial lesions, in particular. Mitochondria generate a large proportion of the reactive oxygen species produced, but these organelles are also their principal victim, due to the disruption of membrane exchanges, leading to a decrease in energetic ATP production. These processes are known to induce the death of the cell by necrosis or apoptosis [31]. These events have already been described in experimental studies in various animal models of uterine ischemia. In a UTx model in ewes, Wranning et al. showed that modulation of the nature of the solution used for graft storage could reduce the oxidative stress generated during reperfusion. Indeed, the use of Perfadex,^®^ rather than Ringer’s acetate solution prevented the increase in oxidative stress markers, such as the plasma concentration of ascorbyl radicals or of thiobarbituric acid reactive substances, within the first few hours of reperfusion [13]. Oxidative stress results from an imbalance between the pro- and antioxidant factors in favor of the pro-oxidant factors. Glutathione is considered to be a cellular redox buffer and, thus, a major antioxidant. It is naturally present in the body and protects cell integrity by neutralizing the ROS produced during various cellular metabolic processes and during ischemia-reperfusion sequences. The nature of the storage solution may also modulate this antioxidant defense system in tissues. Wranning et al. compared various storage solutions for human uteri (Ringer’s lactate solution, UW and Perfadex^®^) after a short period of CI. They showed, in particular, that uterine tissues stored in UW for 6 or 24 h had higher intratissue concentrations of total glutathione than the tissues of other groups [32]. In a model of combined ischemia-reperfusion of the ovaries and uterus in rats, Aslan et al. observed a significant decrease in the levels of glutathione and superoxide dismutase 90 min after reperfusion. Superoxide dismutase is an antioxidant enzyme responsible for eliminating a highly ROS, the superoxide anion, which is associated with a significant increase in lipid peroxidation, as demonstrated by measurements of the intratissue concentration of malondialdehyde [33]. These results suggest that oxidative stress is a process engendered by the uterine ischemia-reperfusion sequence, and that classical markers can be used to evaluate its intensity. Ischemia leads to a cessation of blood flow, stopping the oxygen supply, thereby inhibiting mitochondrial oxidative phosphorylation. This leads to a decrease in ATP production, with ATP subsequently produced mainly via anaerobic metabolism, which is characterized by the production of lactate. There may be a rapid reversion to aerobic metabolism during the reintroduction of the oxygen supply during reperfusion. In a model of autologous UTx in ewes, Dahm-Kähler et al. showed that plasma lactate concentration decreased progressively and rapidly, within less than 60 min of reperfusion [12]. It has also been suggested that lactate may act as a valuable energetic substrate for hepatic neoglucogenesis during situations of energetic crisis, such as ischemia-reperfusion. The decrease in oxygen supply is also linked to the induction of adaptation processes. The best known is that mediated by the transcription factor HIF1a, its intracellular stability is sensitive to the partial pressure of oxygen. In 2017, Kwiatkowska et al. [34] showed, in a porcine model of 60 min of uterine ischemia induced by arterial ligation, that HIF1a expression increased in the endometrium, confirming a tissue response to hypoxia within this organ. However, they also showed that uterine regions have different sensitivities to hypoxia.

All these pathophysiological processes are associated with an often-deleterious inflammatory process during uterine ischemia-reperfusion. The actors in this process remain unclear and their further characterization is therefore required. To this end, Okazaki et al. [35] highlighted the essential role of TNF-α, a pro-inflammatory marker, in the induction of apoptosis by the ischemia-reperfusion sequence in a mouse model of uterus horn and uterine artery clamping. Saso et al. recently investigated changes in oxygen saturation in the uterus in the context of transplantation in rabbits and ewes, by pulse oximetry and multispectral imaging (MSI) [36]. They also showed that oxygen saturation levels decreased significantly after transplantation relative to the rates measured before graft procurement and transplantation.

Our team has recently performed 18 autologous UTx in ewes. We studied the metabolic changes induced by a 24 h CI. For this, we analyzed samples of Celsior^®^ with a dual approach: targeted biochemical analyzes targeting several predefined metabolites, and non-targeted metabolic analyzes based on nuclear magnetic resonance (NMR). The metabolic results indicate a significant degradation of the uterus during 24 h of CI. This organ tolerates storage periods of this length poorly, as shown by the increase in the levels of markers of cell lysis, acidosis and oxidative stress. The metabolic analysis obtained from preservation solution samples during CI of the graft could become a predictive tool to assess uterine injury. These markers could be used as reproducible outcome measures for assessing the uterine response to CI and useful for future comparative studies [37].

#### 4.1.5. Ways of Increasing Uterine Tolerance to CI or WI

One recent study focused on the possibility of improving uterine CI tolerance by adding an antioxidant, acetyl L-carnitine, to the storage fluid [38]. Rat uteri (*n* = 24) were stored for 4 or 24 h in histidine-tryptophan-ketoglutarate (HTK) solution, with or without acetyl L-carnitine (4 groups). Histological analysis revealed signs of uterine alteration after 24 h of storage without the addition of acetyl L-carnitine. For similar CI times, lower levels of VEGFR-2 were found in the groups with acetyl L-carnitine than in those without the antioxidant. VEGFR-2 is secreted in response to hypoxia, hypoglycemia, inflammation, and is involved in tissue repair. The authors therefore concluded that the addition of acetyl L-carnitine to the HTK storage fluid prevented the formation of free radicals during CI, thereby protecting the uterus. Barun et al. performed a similar study in 2011. Their experimental model was identical except that the adjuvant added to HTK was iloprost, a prostacyclin analog with antioxidant properties. They found no significant difference between groups in terms of malondialdehyde (MDA) and nitric oxide levels, these two molecules being used as markers of oxidative stress. Histological analyses revealed uterine alterations after 24 h of CI, and these alterations were attenuated by the addition of iloprost to the HTK [39].

Other studies have investigated the possibility of prolonging uterine tolerance to WI with the aid of pharmacological adjuvants. The animal model used for all these studies was the rat. Ischemia was induced by arterial clamping. In 2014, Sahin et al. [40] reported the results of a prospective study, in which premedication with tacrolimus in 28 rats protected against the appearance of oxidative stress in the uterine, this major phenomenon being induced by the ischemia-reperfusion sequence, through the inhibition of lipid peroxidation and the stimulation of antioxidant defenses. Sahin Ersoy et al. [41] investigated another immunosuppressant, mycophenolate mofetil (MMF). They found that MMF had a protective effect against the lesions induced by ischemia-reperfusion, mediated by a cytostatic effect on lymphocytes and on the expression of adhesion molecules. Following pretreatment with MMF in a similar animal model, they observed significantly lower levels of histological lesions and of apoptosis and markers of oxidative stress (8-hydroxy-2′-deoxyguanosine (8-OHdG), MDA, myeloperoxidase (MPO)) 4 h after reperfusion. These results are consistent with a protective effect of MMF, attenuating the tissue lesions induced by uterine ischemia-reperfusion. In 2017, Aslan et al. [33] investigated the anti-inflammatory and antioxidant properties of oxytocin and kisspeptin, two proteins involved in the control of reproductive functions and puberty. They reported significantly lower levels of histological alteration and oxidative stress markers following premedication with oxytocin and kisspeptin before arterial clamping in 24 rats. In 2015, Atalay et al. [42] concluded that the continuous administration of remifentanil during ischemia-reperfusion had a protective effect against the extension of histological lesions of the uterus, by decreasing the concentration of infiltrating leukocytes and cell degeneration. They also showed that the generation of oxidative stress was limited by a significant decrease in MDA concentrations and an increase in the activity of antioxidant enzymes, such as catalase and superoxide dismutase, in uterine samples, in a rat model involving 2 h of uterine WI followed by one hour of reperfusion.

In 2010, Dittrich et al. investigated the possibility of cryopreserving uteri from sows. Following hysterectomy and cannulation of the uterine arteries, the uteri were perfused with a cryoprotective agent based on dimethyl sulfoxide (DMSO) and progressively frozen down to −150 °C. Sixteen weeks later, they were rapidly warmed. The authors found that 100% of the uteri were contractile after the injection of oxytocin if the cryoprotective fluid contained 5% or 10% DMSO. By contrast, only 40% of uteri were contractile for 1% DMSO and none were contractile for 0.5% DMSO. No histological alterations to the uterus were detected when 5% or 10% DMSO was added to the cryoprotective solution [43].

Overall, the use of antioxidant or pharmacological adjuvants improved ischemia tolerance in the uterus. However, to date, these studies have not progressed beyond basic research and have to be validated at a clinical level.

### 4.2. Human Studies

#### 4.2.1. Impact of CI in the Absence of Reperfusion

In physiological conditions, body temperature is kept constant in mammals, which are therefore classified as homeothermic. CI greatly decreases cellular metabolism and energy needs during graft storage. However, organs are not adapted to deal with low temperatures of the order of 4 °C. This ex vivo storage of organs in hypothermic conditions is not without consequences, due partly to the ischemia, resulting in a lack of oxygen and energetic substrate, but also due to the hypothermia itself, with prolonged exposure to a temperature of 4 °C for several hours.

Some studies have tried to determine the maximum duration of human uterine tolerance to CI. These studies include that of Del Priore et al. in 2007 [44] and our own study in 2014 [45]. These two prospective studies evaluated the histological and immunohistochemical modifications occurring after different CI times, after the procurement of human uteri in a context of multiorgan procurement (MOP) without reperfusion. Del Priore observed no histological modifications on electron microscopy after 12 h of CI and storage in a solution of the UW type [44]. We observed no histological changes or increases in apoptosis, as assessed in TUNEL assays, after 24 h of CI and storage in Celsior^®^ solution [45]. In 2008, Sieunarine et al. [46] studied the histological modifications occurring in human uterus specimens after 24 and 48 h of storage in Celsior^®^ storage solution. They observed no major histological change after 48 h of CI, and even reported the myoendometrium to be perfectly intact after 24 h of storage. In 2005, Wranning et al. [32] also studied changes of some biological parameters of energy metabolism (ATP) and oxidative stress (glutathione), together with the capacity of muscle cells to contract after various CI times, in samples of human uteri obtained during hysterectomies for benign conditions. They also compared different organ storage fluids (UW and Perfadex^®^). They observed a decrease in spontaneous contraction capacity, regardless of the duration of CI, in all groups. However, spontaneous uterine contraction and contraction after stimulation with la PGF_2α_ at 6 h of CI were better conserved in the UW and Perfadex^®^ groups. No histological modification was observed on electron microscopy, but a degeneration of the vesicular cytoplasm and chromatin condensation were observed after 24 h of CI in Ringer’s acetate solution. The concentration of ATP was significantly higher in tissues stored in Perfadex^®^ or UW than in tissues stored in Ringer’s acetate solution. Wranning et al. concluded that their results showed that the uterus could tolerate 6 h of CI if stored in UW or Perfadex^®^.

Overall, analyses of uterine fragments after prolonged CI showed promising results; the uterus could maintain its contractile function and histological characteristics after at least 6 h of CI.

#### 4.2.2. UTx in Women

Worldwide, at least 52 uterus transplants have been performed in women, according to the first state-of-the-art meeting on UTx, which took place at Ghent in October 2018 [5]. Several UTx have also been published in the press, notably the first UTx with a live donor performed in France (March 2019, Paris). All procedures published through scientific publications are described in Table 2 [2,3,47,48,49,50,51,52,53,54,55,56,57,58,59,60,61]. Ten failures of transplantations have been reported in publications: one in the Saudi Arabia [2]; two by the Swedish team of Pr. Brännström [3]; three by the Dallas team (Testa et al.) [53,54]; one by the Cleveland team (Flyckt et al.) [52]; and three in Czech Republic [61]. The failure of UTx being defined by the need to explant the graft before the embryo transfer. To date, 13 of UTx have led to live births of children in good health, corresponding to 25% (13/52) of pregnancies obtained by UTx [5]. The first pregnancy was obtained in 2011, by a Turkish team. Unfortunately, the pregnancy self-terminated at seven weeks of gestation, requiring fetus removal by aspiration [48,49]. In 2014, the Swedish team of Pr. Brännström performed a series of nine UTx. Seven of the grafts were viable and allowed the birth of eight healthy children between 2014 and 2017 [3,56]. Then, in 2017, the American team from Dallas performed a series of five transplantations. The grafts were viable in two of these cases, resulting in two pregnancies and the births of two healthy children [53,54]. The first birth after UTx from a brain-dead donor occurred in Brazil [60]. The last birth—to be reported in the media after UTx—was obtained in Serbia after the first UTx, between two monozygotic twins and in India. In the latter, it was the first baby born in a case of non-absolute uterine infertility.

There are two types of donors for UTx: living donors and brain-dead donors. Six teams work only with living donors [2,3,50,56,58] (Saudi Arabia, Sweden, China, Germany, Serbia and India) and three works solely with brain-dead donors [48,52,55] (Turkey, Cleveland and Brazil). There are also two teams working with both types of donors [61,62] (Dallas and Czech Republic).

There is currently no consensus concerning the best storage fluid. Most teams use an HTK solution [3,50,53,58], in Sweden (Brännström et al.), China (Wei et al.), Dallas (Testa et al.), Germany (Brücker et al.) and Brazil. In Saudi Arabia, a Euro-Collins solution has been used [2] and the Turkish team uses UW solution [48].

UTx surgery is a difficult technique, necessitating the coordination of two teams. Major surgical and ischemia time data are summarized in Table 2. Overall, most of the births occurring after UTx from a living donor have therefore occurred after only moderate periods of ischemia. However, the data for the first birth following UTx from a brain-dead donor and a CI time of more than 6 h in Brazil are promising.

## 5. Discussion

Published data for UTx show that the human uterus can tolerate a period of CI up to 6 h at least, permitting a favorable pregnancy outcome [60]. Before the development of UTx, studies of uterine embolization, particularly in cases of post-partum hemorrhage, showed that the uterus could tolerate a transient period of ischemia, with the subsequent achievement of pregnancies, and no consequences for fertility [63,64]. In the framework of UTx, data from animals also seem to indicate a high tolerance to ischemia. Indeed, pregnancies have been obtained after 24 h of CI in mice [8]. By contrast, most of the studies in large animals were performed by teams planning to perform UTx from live donors, accounting for the very short periods of CI in these studies. Only one study demonstrated the occurrence of uterine contractions after 24 h of CI [16]. It is, therefore, currently difficult to define a threshold, particularly for CI time, beyond which the uterus is unlikely to be functional. Further studies are therefore required, in large animals, with extreme CI times, to define this threshold. In humans, studies on UTx from brain-dead donors, for which CI times may be long, are required to enable us to evaluate the tolerance of the human uterus to ischemia. To date, only one live birth has ever been achieved after UTx from a brain-dead donor, in Brazil [60]. It has also been demonstrated that the composition of the storage solution has an impact on the re-establishment of graft function and the best universal solution for all organs has yet to be determined. Some solutions, such as UW or SCOT^®^, are more suitable for kidney grafts, whereas others, such as Celsior^®^ are more appropriate for hearts. The success rate for the transplantation of uteri stored in HTK solution, which was initially used for heart grafts, suggests that this solution is suitable for UTx [3,50,53,54,58,60]. HTK, an extracellular solution, is characterized by low concentrations of sodium and potassium, whereas UW and Euro-Collins solutions, which have also been used for UTx in humans, have higher potassium concentrations and potentially deleterious effects on the graft [65]. In addition, a study by Ugurlu et al. [38] shows that the protective effect of a storage fluid can be improved further by adding pharmacological agents, which may make it possible to increase the storage time of organs. However, other comparative studies are required to determine which storage fluid is the most appropriate for UTx. The knowledge of metabolic biomarkers as, judgement criterion, could be a tool to compare and to choose the best storage solution. These biomarkers could be identified using new research techniques, such as metabolomics, as already done for vital organs [37,66].

In kidney transplantation, hypothermic perfusion machines, providing a continuous supply of metabolic substrates and maintaining microvascularization during CI, have proved to be effective during the storage of organs in CI; reducing the damage caused to the organ [67,68]. An ex vivo uterus reperfusion platform could be an interesting way in UTx to decrease ischemia- and reperfusion injury [17]. Similarly, the use of hemoglobin from the marine worm *Arenicola marina,* which has an enhanced affinity for O_2_, has also proved to be effective during CI for lung and kidney transplantation [69,70,71]. These approaches could be applied to UTx.

During the second phase of WI, it should be possible to improve uterine tolerance through the use of pharmacological agents, as shown by several studies [33,40,41,42]. By contrast, only training and practice in the procedures for vascular anastomosis can decrease the duration of WI. The results of the Dallas team indicate the existence of such a learning curve, because their WI times decreased and their success rates for UTx increased with the number of procedures performed [53]. The development of new anastomal techniques using the utero-ovarian veins for drainage and the internal iliac arteries for perfusion seem to facilitate the surgical procedure and reduce the operating time and therefore the ischemia time [59,72,73].

To date, worldwide, more than 50 UTx have been performed in humans. The increasing number of procedures performed will add to the data available and improve our knowledge of uterine ischemia tolerance, making it possible to modify protocols to maximize the chances of UTx success. To this end, a register compiling all cases of UTx performed worldwide has been set up by the International Society for Uterus Transplantation (ISTUx).

## Figures and Tables

**Table 1:** Summary of the studies in which live births have been obtained after uterus transplantation (UTx) in animals.

Study	Year	Animal Species	Type of Transplantation	Maximum Duration of Cold Ischemia (min)	Maximum Duration of Warm Ischemia (min)	Maximum Total Ischemia Time (min)	Number of Live Births/Number of Successful UTx
El-Akouri et al. [8]	2003	Mouse	Syngeneic	1440	NC	NC	25/6
El-Akouri et al. [9]	2003	Mouse	Syngeneic	20	NC	NC	12/12
Wranning et al. [10]	2011	Rat	Syngeneic	120	NC	NC	6/19
Diaz-Garcia et al. [25]	2014	Rat	Allotransplantation	NC	NC	NC	NC/14
Wranning et al. [14]	2010	Ewe	Autologous transplantation	60	NC	NC	2/7
Ramirez et al. [28]	2010	Ewe	Allologous transplantation	60	45	NC	1/12
Mihara M et al. [21]	2012	Cynomolgus macaque	Autologous transplantation	NC	285	427	1/2

NC = not communicated.

**Table 2:** Summary of UTx published, by type of donor, type of surgery and graft failure.

	Country (Number of Reported UTx)	Pathological Condition of the Recipient	Brain-Dead (BD) or Living Donor	Surgical Approach	Mean Duration of Surgery for Donor (±SD) (min)	Mean Duration of Surgery Recipient (±SD) (min)	Cold Ischemia Time (±SD) (min)	Warm Ischemia Time (±SD) (min)	Storage Solution Used	Graft Survival (Number)	Pregnancies (Number)	Births (Number)
2000	Saudi Arabia (1) [2]	Hysterectomy for PPH	Living	Laparotomy	NC	NC	NC	NC	Euro-Collins	Necrosis leading to explantation on day 99	No	No
2011	Turkey (1) [48,49]	MRKH	BD	Laparotomy	NC	NC	NC	NC	UW	Yes (1)	Yes (5)	No
2012 2013	Sweden (9) [3,4,51]	Hysterectomy Cervical cancer (1) MRKH (8)	Living	Laparotomy	697 (±65)	286 (±30)	78 (±23)	83 (±9)	HTK	2 explantations: 1 uterine artery thrombosis and 1 pelvic abscess, 7 viable	Yes (8)	Yes (8)
2015	China Xian (1) [58]	MRKH	Living	Robot-assisted surgery (donor) Laparotomy (recipient)	360	530	213	89	HTK	Yes (1)	NC	NC
2015	United States Dallas (5) [53,54]	MRKH	Living	Laparotomy	492 (±27)	318 (±46)	210 (±42)	62 (±13)	HTK	3 explantations: 2 uterine artery thromboses 2 viable	Yes (2)	Yes (2)
2016	United States Cleveland (1) [52]	MRKH	BD	Laparotomy	NC	NC	NC	NC	NC	No (1) Fungal infection	No	No
2016	Czech Republic (9) [61]	MRKH	Living (5) BD (4)	Laparotomy	356 (±41) NC	236 (±33) 258 (±25)	320 (±57) 302 (±145)	NC	NC	Yes (6) 3 explantations: 2 uterine artery thrombosesAnd one HSV-2 infection	No	No
2016	Brazil (1) [60]	NC	BD	Laparotomy	NC	630	380	90	HTK	Yes (1)	Yes (1)	Yes (1)
2017	Germany (2) [50]	MRKH	Living	Laparotomy	635 (±127)	314 (±61)	NC	NC	HTK	1 uterus not transplanted (peroperative atherosclerosis) 2 viable	NC	No
2017	Sweden (2) [56,57]	NC	Living	Robot-assisted (donor)	NC	NC	NC	NC	NC	Yes (2)	NC	NC
Total	32										16	11

SD: standard deviation; PPH: Post partum hemorrhage; MRKH: Mayer-Rokitansky-Küster-Hauser syndrome; NA: not applicable; NC: not communicated.
